# A bivalent glycopeptide to target two putative carbohydrate binding sites on FimH

**DOI:** 10.3762/bjoc.6.90

**Published:** 2010-08-24

**Authors:** Thisbe K Lindhorst, Kathrin Bruegge, Andreas Fuchs, Oliver Sperling

**Affiliations:** 1 Christiana Albertina University of Kiel, Otto Diels Institute of Organic Chemistry, Otto-Hahn-Platz 4, D-24098 Kiel, Germany, Fax: +49 431 8807410

**Keywords:** bacterial adhesion, bivalent ligand, ELISA, FimH, glycopeptides

## Abstract

FimH is a mannose-specific bacterial lectin found on type 1 fimbriae with a monovalent carbohydrate recognition domain (CRD) that is known from X-ray studies. However, binding studies with multivalent ligands have suggested an additional carbohydrate-binding site on this protein. In order to prove this hypothesis, a bivalent glycopeptide ligand with the capacity to bridge two putative carbohydrate binding sites on FimH was designed and synthesized. Anti-adhesion assays with the new bivalent ligand and type 1-fimbriated bacteria have revealed, that verification of the number of carbohydrate binding sites on FimH with a tailor-made bivalent glycopeptide requires further investigation to be conclusive.

## Introduction

Bacterial adhesion is a phenomenon which occurs on the surface of host cells as well as on the surface of surgical implants, where it can lead to the formation of persistent biofilms. In all cases of bacterial adhesion and of biofilm formation severe health problems can result for the host organism [1–2]. A number of microbial adhesins are known, that co-operate in the adhesion process [3], such as the fimbriae, which are long filamentous adhesive organells on the surface of many bacteria, comprising carbohydrate-binding sub-units [4–6]. The type 1 fimbriae, for example, which are widely spread among the Enterobacteriaceae are terminated with the mannose-specific protein FimH. FimH is structured in the form of two domains, a carbohydrate-specific adhesin domain and a pilin domain, which is required for fimbriae assembly [7]. The FimH adhesin domain features a carbohydrate binding site at its tip, called the carbohydrate recognition domain (CRD), which is known from X-ray studies [8–11]. It is a monovalent binding site, which can accommodate one α-D-mannosyl moiety, for example, the terminal mannoside residues of high-mannose-type glycoproteins of the glycocalyx [12]. However, the precise nature of the ligand-receptor interactions is not fully understood. For example, when multivalent carbohydrate ligands were tested as ligands of FimH and type 1 fimbriated bacteria, respectively [13–14], multivalency effects were observed in many cases. In addition, concentration-dependent inhibitory and stimulating allosteric effects on adhesion have also been reported with certain carbohydrate ligands [15], which might be explained by the existence of allosteric binding sites.

Thus, it has been suggested that there could be multiple binding sites on the FimH adhesin [16] but this hypothesis has so far neither been proven nor disproven. It is interesting to note, that although the sequence of the FimH adhesin is highly conserved, studies by Sokurenko and collegues [17–20] have indicated that allelic variation in FimH is correlated with different carbohydrate-binding profiles. None of the allelic variations giving rise to differences in mannose-binding occurs within, or even close to, the FimH mannose-binding pocket. Additional sugar-binding sites dispersed throughout the lectin domain are a possible explanation for this finding. This feature could aid in recognising large and multivalent carbohydrate receptors respectively, on the host surface.

**Figure 1:**
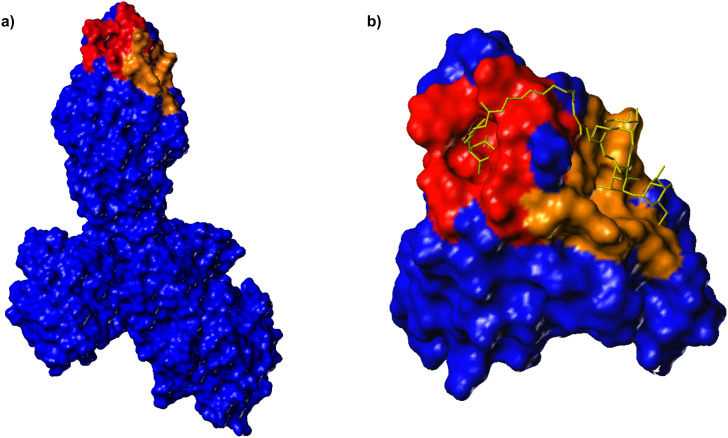
**a)** Connolly surface of FimH in complex with FimC [8]. The CRD known from X-ray structures at the tip of FimH is coloured in red and can accommodate one α-D-mannosyl residue. A second hypothetical carbohydrate binding region on the protein, as suggested by modeling studies [16], is coloured in brown and represents a more extended area on the protein. **b)** Docking studies were used to estimate the length of a linker that is required to bridge the putative two binding sites.

In order to look for possible additional carbohydrate-binding sites in the FimH lectin, the surface of the lectin domain was probed by computational docking studies [16]. Three new potential carbohydrate binding cavities on the surface of the FimH lectin domain, in addition to the mannose pocket at the tip of the domain, were identified which have a marked preference for the same subset of high-mannose trisaccharide substructures, mainly α-D-Man-(1→3)-[α-D-Man-(1→6)]-D-Man. By employing site directed mutagenesis, it was found that mutations in one of these cavities significantly reduces binding, indicating that this could be a second carbohydrate binding site, relevant for ligand binding [21]. Thus, it was our goal to design a bivalent carbohydrate ligand so shaped that it could concomitantly occupy the mannose binding site at the tip of the adhesin domain and the putative second carbohydrate binding site on the receptor. Based on the published structure of FimH, we have estimated the distance between the known CRD at the tip of FimH and the suggested second binding site, which is a more extended region on the protein (Figure 1). Docking using FlexX [22–24] recommended a spacer of 10 to 15 amino acids to ligate the two different carbohydrate ligand portions of a bivalent glycoconjugate. This corresponds to a linker length of between 30 and 40 Å. While the known CRD accommodates exactly one α-D-mannosyl residue in the binding pocket, with the aglycone of the mannoside sticking out of the binding site, the postulated second binding site could rather interact with a carbohydrate of the size of a mannotrioside. Hence, a monomeric mannoside and the trisaccharide α-D-Man-(1→3)-[α-D-Man-(1→6)]-D-Man were selected as carbohydrate ligands and azidoethyl aglycone moieties were chosen to allow their ligation via an oligoglycine spacer of an appropriate length.

**Figure 2:**
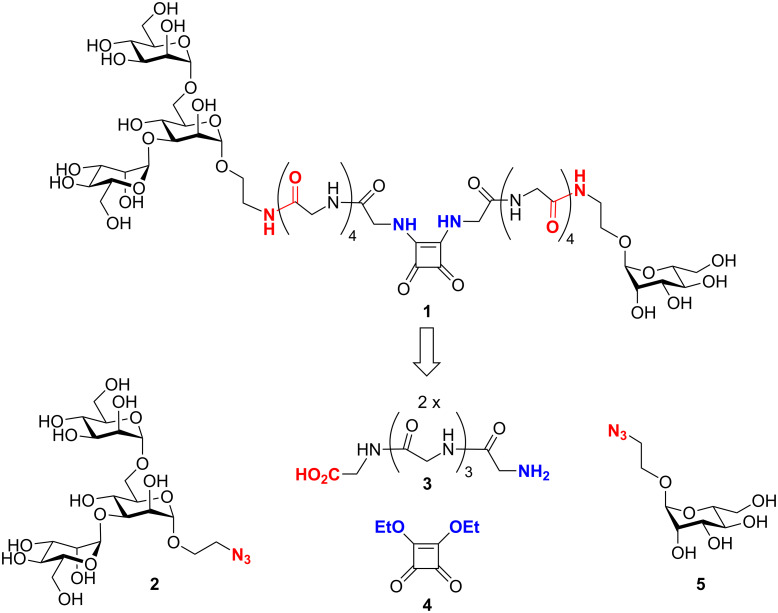
The bivalent glycopeptide **1** is the target molecule to test the hypothesis of two carbohydrate binding sites on FimH. Its retrosynthesis delivers the azido-functionalized mannotrioside **2** as the western part of the target structure and 2-azidoethyl mannoside **5** as its eastern portion. Squaric acid diethyl ester (DES, **4**) can link both parts via two pentaglycine spacers (**3**).

The well-known squaric acid diester linkage strategy [25] was applied to connect the monosaccharide and the trisaccharide part of the bivalent glycopeptide target structure **1** (Figure 2). Accordingly, retrosynthetic analysis of **1** leads to the 2-azidoethyl glycosides **2** and **5**, with the azido group masking an amino function; two pentaglycine spacer molecules (**3**) and squaric acid diethyl ester (**4**, DES). The synthetic assembly relies on peptide coupling chemistry and the squaric acid diester to link two different amines in two subsequent steps.

## Results and Discussion

**Scheme 1:**
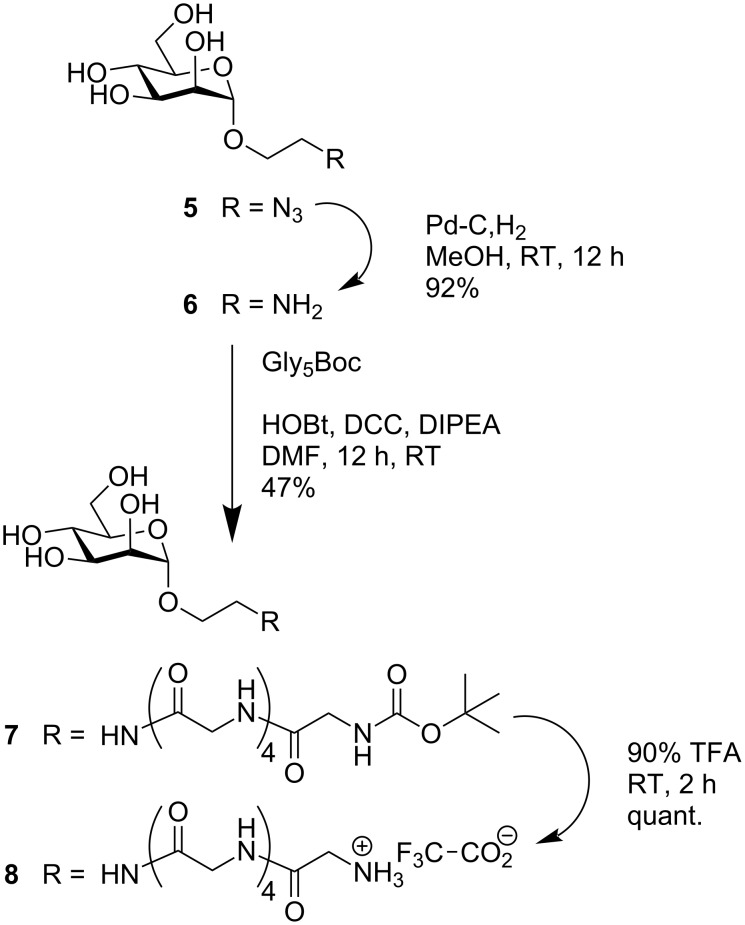
Synthesis of the eastern part of target molecule **1**.

Synthesis of the eastern part of target molecule **1** started from the known azidoethyl mannoside **5**, which can be prepared from mannose pentaacetate in three simple steps [26]. Catalytic hydrogenation led to the amine **6** [27], which was subjected to peptide coupling with *N*-Boc-protected pentaglycine (Gly_5_Boc) under standard reaction conditions (Scheme 1). This led to the *N*-Boc-protected glycopeptide **7** and removal of the Boc protecting group with TFA gave amine **8** as its TFA salt.

**Scheme 2:**
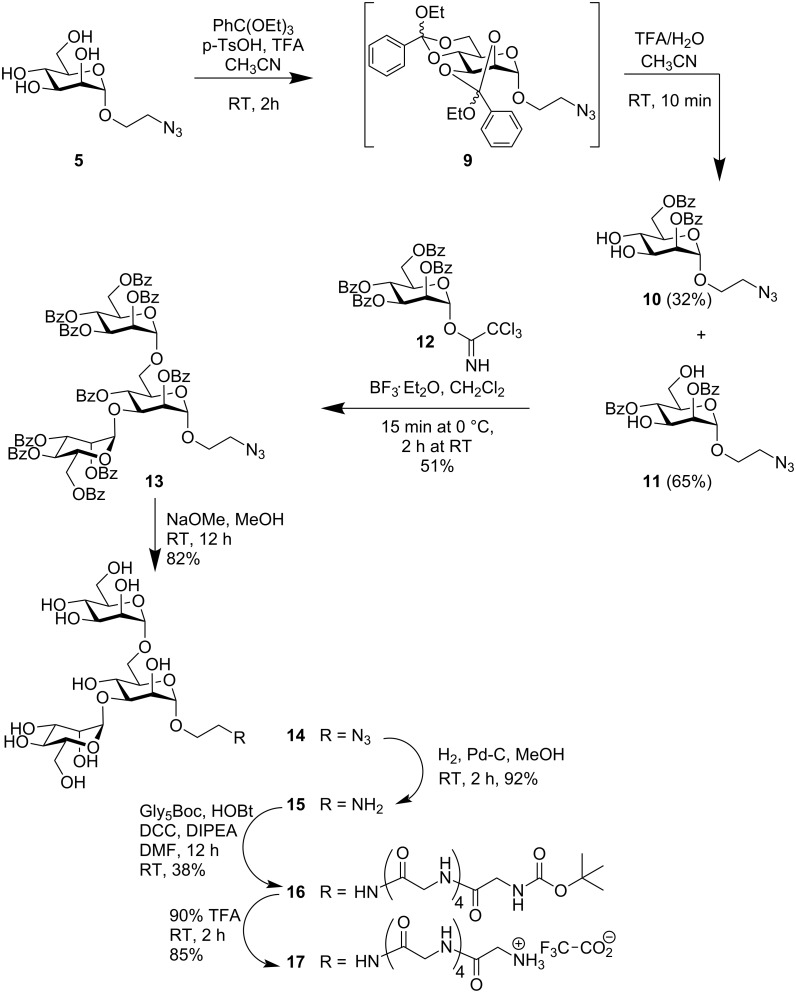
Synthesis of the western part of target molecule **1**.

To prepare the western part of target molecule **1**, the trisaccharide azide **2** was required by analogy to the synthetic pathway leading to glycopeptide **8**. To establish the protecting group pattern that is required for 3,6-bis-mannosylation, the azidoethyl mannoside **5** was converted into the bis-orthoester **9**, which was further converted in situ under acidic conditions to yield the required 2,4-dibenzoate **10** together with the isomeric 2,6-dibenzoate **11** as a byproduct, according to a known literature procedure (Scheme 2) [28–29].

Glycosylation of the acceptor diol **11** with the trichloroacetimidate **12** [30] led to mannotrioside **13** with the required azidoethyl aglycone. Reduction of the azido group gave amine **15** and subsequent peptide coupling reaction with Gly_5_Boc with inexpensive coupling reagents led to the glycopeptide **16**. Boc-deprotection gave the required mannotrioside peptide **17** in the form of its TFA salt.

**Scheme 3:**
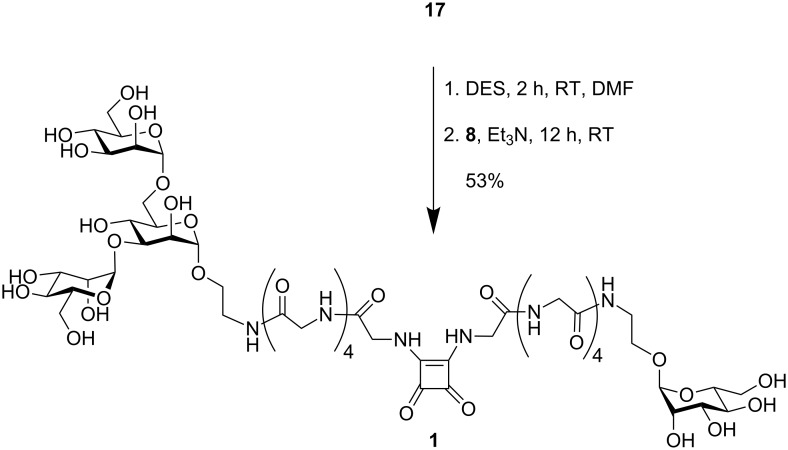
Synthesis of the target molecule **1** employing squaric acid diethylester (DES).

The glycopeptides **8** and **17** were finally ligated via squaric acid employing DES for ligation (Scheme 3). Control of the pH-value during ligation allows sequential substitution [31]: the first amine reacts at neutral pH, whereas addition of base is required to couple a second amine to the intermediate squaric acid monoester. Thus, the bivalent glycopeptide **1** was obtained in pure form, albeit after laborious gel permeation chromatography.

### 

#### Testing of the bivalent glycopeptide 1 in type 1 fimbriae-mediated bacterial adhesion

The synthesis of the bivalent glycopeptide **1** was not optimized in order to improve all the individual yields, because it has been the primary goal of this study to test the anti-adhesive properties of this type of ligand. An ELISA was performed as reported earlier [32–33], employing type 1 fimbriated *E. coli* bacteria and mannan-coated microtiter plates. In this assay a mannosidic ligand competes with the polysaccharide mannan adsorbed on a polystyrene microtiter plate for FimH-mediated binding to the type 1 fimbriated bacteria. The inhibitory ligand is employed in serial dilutions. This leads to inhibition curves, from which IC_50_-values can be deduced. The IC_50_-values reﬂect the inhibitor concentration that causes 50% inhibition of bacterial binding to mannan. ELISA measurements typically lead to IC_50_-values covering a broad range of absolute values. Therefore, highly reproducible relative inhibitory potencies (RIP-values) are usually reported in order to compare different inhibitors. Here, RIPs were based on the IC_50_-value determined for MeMan on the same plate, which was defined as IP ≡ 1. In addition to the bivalent glycopeptide **1**, the disaccharides allyl 3-*O*-α-D-mannosyl-α-D-mannoside (α-D-Man-(1→3)-D-Man-α-allyl) (Table 1, entry 3) and allyl 6-*O*-α-D-mannosyl-α-D-mannoside (α-D-Man-(1→6)-D-Man-α-allyl) (Table 1, entry 4) as well as the branched mannotrioside allyl 3,6-di-*O*-(α-D-mannosyl)-α-D-mannoside (α-D-Man-(1→3)-[α-D-Man-(1→6)]-D-Man-α-allyl) (Table 1, entry 5) were compared to MeMan, as reported earlier (Table 1) [34].

**Table 1:** Inhibitory potencies in mannose-specific *E. coli* adhesion of the bivalent glycopeptide **1** in comparison with reference ligands, as determined by ELISA. IC_50_-values are average values from three independent assays. *S*: standard deviation; RIP: relative inhibitory potency based on methyl α-D-mannopyranoside (MeMan) with IP(MeMan) ≡ 1.

Entry	Tested Ligand	IC_50_ [µmol] (*S*)	RIP

1	MeMan	2900 (890)	1
2	**1**	1500 (360)	2
3	allyl Man(1,3)Man		7^a^
4	allyl Man(1,6)Man		0.5^a^
5	allyl Man(1,3)[Man(1,6)]Man		20^a^

^a^[34].

## Conclusion

From the results collected in Table 1 it is obvious that the new glycopeptide **1** does not lead to a significant increase in the inhibition of type 1 fimbriae-mediated bacterial adhesion in comparison to the standard inhibitor MeMan. Unexpectedly, the bivalent ligand **1** performs just twice as good as MeMan. Consequently, it can hardly be argued that **1** is bridging two binding sites on FimH, or is executing any cooperative effect. Strikingly, ligand **1** is clearly a weaker ligand than the trisaccharide allyl Man(1,3)[Man(1,6)]Man (Table 1, entry 5), even although it contains the same trisaccharide as a partial structure.

There are several possible explanations for our findings with the new bivalent ligand **1**: i. a., (i) ligand **1** does not have the appropriate dimensions to match the two binding sites; (ii) the entropic penalty, that is experienced by the rather extended structure of **1** upon complexation with FimH leads to overall weak binding (high IC_50_-values); (iii) a coherent conformational disadvantage of ligand **1** turns it into an even worse ligand than the branching mannotrioside Man(1,3)[Man(1,6)]Man; (iv) ligand **1** might bind to a different site or to a different protein of the fimbrial shaft, thus lowering its effective concentration.

Thus, the hypothesis of multiple binding sites on FimH could not be conclusively supported by testing the new bivalent ligand **1**, likewise, neither can our findings be taken as counter-evidence. In light of very recent findings, our observations as well as results with other multivalent ligands, might be well explained by an allosteric model of FimH-mediated adhesion [35]. It has been reported that interdomain allosteric regulation can lead to a catch bond mechanism of adhesion in which the adhesive interaction becomes stronger with increased tensile force. The crystal structure of FimH in its native conformation -integrated into ﬁmbrial tips- revealed that the binding domain of FimH is twisted and compressed by interaction with the pilin domain, thus loosening the adhesin mannose-binding pocket. This leads to a low-afﬁnity state of the protein, whereas upon interaction with mannose, the domains separate and the binding domain untwists and elongates into a tight mannose-binding pocket. It will be important to investigate, how complex multivalent ligands function in this allosteric regulation of the ligand-receptor interaction.

## Experimental

### 

#### Docking studies

Computer-aided modeling to estimate the spacer lengths of a bivalent glycopeptide ligand to allow bridging of two putative binding sites on FimH was carried out using FlexX flexible docking and consensus scoring, implemented in Sybyl 6.8, as previously described [36]. Docking was based on the published X-ray structure of the FimH [8].

#### ELISA

An ELISA protocol was used to determine the IC_50_-value of the bivalent target glycopeptide **1** in comparison with monovalent reference ligands, employing mannan-coated microtiter plates and type 1 fimbriated *E. coli* (HB101pPKL4) [37] as described earlier [34] Optical densities (ODs) were measured on an AMP 400 COM ELISA reader at 405 nm (reference wavelength 492 nm). The percentage inhibition was calculated as {[OD(nI)-OD(I)] × 100 × [OD(nI)]^−1^} (nI: no inhibitor, I: with inhibitor). The IC_50_-values were determined where the sigmoidal fit of a set of measured inhibitions crosses an imaginary 50%-line. F-shaped 96-well microtiter plates from Sarstedt were used, mannan from *Saccharomyces cerversiae* was purchased from Sigma and used in 50 mM aq Na_2_CO_3_ solution (1 mg × ml^−1^; pH 9.6). Peroxidase-conjugated goat anti-rabbit antibody (IgG, H+L) was purchased from Dianova.

#### General

All solvents were distilled prior to use. Commercially available starting materials, reagents and anhydrous DMF were used without further purification unless otherwise noted. Air- and/or moisture-sensitive reactions were carried out under an atmosphere of nitrogen or argon. Thin layer chromatography was performed on silica gel plates (GF 254, Merck). Detection was effected by UV irradiation and subsequent charring with 10% sulfuric acid in EtOH followed by heat treatment. Flash chromatography was performed on silica gel 60 (230–400 mesh, particle size 0.040–0.063 mm, Merck). Preparative MPLC was performed on an apparatus of BÜCHI Labortechnik GmbH using a LiChroprep RP-18 (40–60 µm, Merck) column for reversed-phase silica gel chromatography. Gel permeation chromatography (GPC) was carried out on Sephadex LH-20 or on Biogel P4 (bio-Rad), if not otherwise stated. ^1^H and ^13^C spectra were recorded on a Bruker DRX-500 (500 MHz for ^1^H and 125.76 MHz for ^13^C) instrument with Me_4_Si (δ = 0) as the internal standard. Optical rotations were determined with a Perkin Elmer 241 polarimeter (Na-D-line: λ = 589 nm, length of cell: 1 dm). ESI-MS measurements were recorded on a Mariner ESI-TOF 5280 (Applied Biosystems) instrument and MALDI-MS measurements on a MALDI-TOF-MS-Biflex III (Bruker) instrument.

#### 2-[*N*-(*N^ω^*-*tert*-Butyloxycarbonyl-pentaglycyl)]-amidoethyl α-D-mannopyranoside (7)

Glyc_5_Boc (300 mg, 0.74 mmol) was dissolved in dry DMF (10 mL) and DCC (170 mg, 0.83 mmol), HOBt (110 mg, 0.81 mmol), and DIPEA (100 μL) were added at 0 °C. The reaction mixture was stirred for 30 min and then the amine **6** (150 mg, 0.67 mmol) was added. Stirring was continued at 0 °C for another 30 min and then overnight at RT. The mixture was filtered through celite, the solvent removed in vacuo and the residue purified by MPLC-RP chromatography (MeOH:H_2_O = 1:4 → 2:3) to yield the title compound as a white lyophilisate (190 mg, 0.31 mmol, 47%).

^1^H NMR (500 MHz, D_2_O): δ = 4.78 (d, 1H, *J*_1,2_ = 1.8, H-1), 3.95 (m_c_, 6H, 2 NH-C*H*_2_-CO, manOC*H*_2_CH_2_N), 3.93 (s, 2H, NH-C*H*_2_-CO), 3.87 (m_c_, 1H, *J*_2,3_ = 3.5, H-2), 3.86 (s, 2H, NH-C*H*_2_-CO), 3.82 (m, 2H, manOCH_2_C*H*_2_N), 3.81 (dd, 1H, *J*_6a,6b_ = 12.1 Hz, H-6a), 3.77 (s, 2H, NH-C*H*_2_-CO), 3.72 (m, 1H, *J*_3,4_ = 8.9, H-3), 3.59–3.50 (m, 3H, H-4, H-5, H-6b), 1.39 (s, 9H, C(CH_3_)_3_) ppm; ^13^C NMR (125.76 MHz, D_2_O): δ = 175.96 (NH-*C*(O)O), 174.60, 174.36, 173.74 (5 NH-CH_2_-*C*O), 101.98 (C-1), 84.15 (*C*(CH_3_)_3_), 75.11 (C-5), 72.80 (C-3), 72.32 (C-2), 69.10 (C-4), 68.02 (manOCH_2_*C*H_2_N), 63.25 (C-6), 45.82 (manO*C*H_2_CH_2_N), 44.81 (4 NH-*C*H_2_-CO), 41.27 (NH-*C*H_2_-CO), 29.89 (C(*C*H_3_)_3_) ppm; MALDI-TOF-MS: *m/z* 631.1, [M + Na]^+^ (631.3 calcd. for C_23_H_40_N_6_O_13_ + Na).

#### 2-[*N*-(Pentaglycyl)]-amidoethyl α-D-mannopyranoside hydrotrifluoroacetate (8)

The Boc-protected mannoside **7** (190 mg, 0.31 mmol) was dissolved in acetonitrile (5 mL) and treated with TFA (90% in water, 100 μL) for 2 h at RT. Then the solvent was removed in vacuo, the residue dissolved in water and lyophilized to yield the title compound as a white lyophilisate (193 mg, 0.31 mmol, quant.).

^1^H NMR (500 MHz, D_2_O): δ = 4.79 (d ≈ m, 1H, H-1), 3.97 (m_c_, 2H, NH-C*H*_2_-CO), 3.95 (m_c_, 4H, NH-C*H*_2_-CO, manOC*H*_2_CH_2_N), 3.87, 3.86 (each m_c_, each 2H, 2 NH-C*H*_2_-CO), 3.80–3.38 (m, 10H, H-2, H-3, H-4, H-5, H-6a, H-6b, manOCH_2_C*H*_2_N, NH-C*H*_2_-CO) ppm; MALDI-TOF-MS: *m/z* 531.1 [M + Na]^+^ (531.2 calcd. for the free amine C_18_H_32_N_6_O_11_ + Na).

#### 2-Azidoethyl 2,4-di-*O*-benzoyl-3,6-di-*O*-(2,3,4,6-tetra-*O*-benzoyl-α-D-mannopyranosyl)-α-D-mannopyranoside (13)

The 2,4-di-*O-*benzoyl-protected mannoside **11** [28] (250 mg, 0.55 mmol) and the trichloroacetimidate **12** [30] (950 mg, 1.2 mmol) were dissolved in dichlormethane (20 mL) under an argon atmosphere. Molecular sieves (4 Å, 100 mg) and BF_3_-etherate (200 µL) were added at 0 °C. The reaction mixture was stirred at this temperature for 30 min and then at RT overnight. Water was added (200 µL), the solvent removed and the residue co-distilled with toluene in vacuo. Purification of the residue by GPC on Sephadex LH-20 (MeOH/dichlormethane, 1:1) led to a white amorphous solid (451 mg, 279 mmol, 51%).

^1^H NMR (500 MHz, CDCl_3_): δ = 8.32, 8.16, 7.84, 7.76, 7.72 (each m_c_, 20H, *o*-aryl-H), 7.60–7.10 (m, 30H, *m*- and *p*-aryl-H), 6.10, 6.02, 5.88 (each dd ≈ t, 3H, *J* = 9.9 and 10.0 Hz, H-3, H-3’, H-3’’), 5.74, 5.72, 5.69 (each dd, 3H, *J*_3_ = 3.4 and 3.5 Hz, H-2, H-2’, H-2’’), 5.36 (d, 1H, d, *J*_2_ = 1.7 Hz, H-1), 5.19, 5.15 (each d, 2H, *J* = 1.8 Hz, H-1’, H-1’’), 4.67–4.60 (m_c_, 3H, H-6a, H-6a’, H-6a’’), 4.36 (each dd, 3H, *J* = 9.6, H-6b, H-6b’, H-6b’’), 4.27 (m_c_, 3H, H-4, H-4’, H-4’’) 4.14 (m_c_, 3H, *J* = 10.8 and 10.9 Hz, H-5, H-5’, H-5’’), 3.79 (ddd, 2H, *J* = 5.7 and 9.7 Hz, manOC*H*_2_CH_2_N_3_), 3.54 (ddd, 2H, manOCH_2_C*H*_2_N_3_) ppm; ^13^C NMR (125.76 MHz, CDCl_3_): δ = 166.3–161.7 (10 C=O), 134.4–132.8 (10 CH, *p-a*ryl-C), 131.8–127.6 (20 CH, *o*-aryl-C), 127.2–126.3 (20 CH, *m*-aryl-C), 99.8, 97.5, 97.3 (C-1, C-1’, C-1’’), 73.5-64.5 (C-2, C-2’, C-2’’, C-3, C-3’, C-3’’, C-4, C-4’, C-4’’, C-5, C-5’, C-5’’), 63.3 (manOCH_2_*C*H_2_N_3_), 61.6, 61.4, 61.9 (C-6, C-6’, C-6’’), 48.9 (manO*C*H_2_CH_2_N_3_) ppm; MALDI-TOF-MS: *m/z* 1637.39 [M + Na]^+^ (1636.45 calcd. for C_90_H_75_N_3_O_26_).

#### 2-Azidoethyl 3,6-di-*O*-(α-D-mannopyranosyl)-α-D-mannopyranoside (14)

The protected mannotrioside **13** (400 mg, 0.24 mmol) was dissolved in dry MeOH (20 mL) and treated with sodium methanolate solution (1 M, 400 μL) at RT. After stirring overnight, the reaction mixture was neutralized by addition of ion exchange resin (Amberlite IR-120), filtered, the filtrate evaporated and the residue purified by reversed-phase chromatography on silica gel (H_2_O:MeOH = 1:5) to yield the title compound as a colourless lyophilisate (113 mg, 0.20 mmol, 82%).

^1^H NMR (500 MHz, D_2_O): δ = 5.09, 4.89, 4.87 (each d, 3H, *J* = 1.8 and 1.9 Hz, H-1, H-1’, H-1’’), 4.12, 4.05, 3.98 (each dd, 3H, *J*_3_ = 3.0, 3.4, and 3.5 Hz, H-2, H-2’, H-2’’), 3.87 (m_c_, 9H, H-3, H-3’, H-3’’, H-4*, H-4’*, H-5, H-6a,* H-6b*, H-6b’*), 3.73 (m_c_, 5H, H-5’, H-5’’, H-6a’*, H-6’’*, manOC*H*_2_CH_2_N_3_), 3.65 (m_c_, 3H, H-4’’*, H-6b’’*) 3.51 (2 H, m_c_, manOCH_2_C*H*_2_N_3_) ppm; ^13^C NMR (125.76 MHz, D_2_O): δ = 104.1, 101.33, 100.73 (C-1, C-1’, C-1’’), 74.98, 74.93, 74.39, 73.76, 72.68, 72.47, 72.13, 71.39, 68.79, 68.53, 67.51, 67.32 (C-2, C-2’, C-2’’, C-3, C-3’, C-3’’, C-4, C-4’, C-4’’, C-5, C-5’, C-5’’), 66.7 (manOCH_2_*C*H_2_N_3_), 65.8, 64.3, 64.1 (3 C-6), 51.3 (manO*C*H_2_CH_2_N_3_) ppm; assignments indexed with * are interchangable. MALDI-TOF-MS: *m/z* 595.88 [M + Na]^+^ (596.19 calcd. for C_20_H_35_N_3_O_16_ + Na).

#### 2-Aminoethyl 3,6-di-*O*-(α-D-mannopyranosyl)-α-D-mannopyranoside (15)

The azide **14** (100 mg, 0.17 mmol) was dissolved in dry methanol (10 mL) and Pd on charcoal (10%, 10 mg) added. Hydrogenation with vigorous stirring for 3 h led to the title amine, which was obtained after filtration through celite, evaporation and reversed-phase chromatography on silica gel (H_2_O:MeOH = 1:1) as a colourless lyophilisate (86 mg, 0.16 mmol, 92%).

^1^H NMR (500 MHz, D_2_O): δ = 5.05, 4.82, 4.79 (each d, 3H, *J*_1,2_ = 1.7, 1.8, and 1.9 Hz, H-1, H-1’, H-1’’), 4.03, 3.96, 3.89 (each dd, 3H, *J_3_* = 3.0 and 3.4, H-2, H-2’, H-2’’), 3.83–3.71 (m, 9H, H-3, H-3’, H-3’’, H-5, H-5’, H-5’’, H-6a, H-6a’, H-6a’’), 3.68–3.60 (m, 6H, H-4, H-4’, H-4’’, H-6b, H-6b’, H-6b’’), 3.40 (m_c_, 4H, manOC*H*_2_C*H*_2_NH_2_) ppm; ^13^C NMR (125.76 MHz, D_2_O): δ = 104.0, 102.3, 101.9 (C-1, C-1’, C-1’’), 83.2, 82.9, 82.7, 81.6, 79.9, 78.6, 78.2, 75.3, 74.9, 74.8, 72.8, 71.7 (C-2, C-2’, C-2’’, C-3, C-3’, C-3’’, C-4, C-4’, C-4’’, C-5, C-5’, C-5’’), 70.2 (manOCH_2_*C*H_2_NH_2_), 69.7, 67.6, 67.4 (C-6, C-6’, C-6’’), 52.3 (manO*C*H_2_CH_2_NH_2_) ppm; MALDI-TOF-MS: *m/z* 570.24 [M + Na]^+^ (570.20 calcd. for C_20_H_37_NO_16_ + Na).

#### 2-[*N*-(*N^ω^*-*tert*-Butyloxycarbonyl-pentaglycyl)]-amidoethyl 3,6-di-*O*-(α-D-mannopyranosyl)-α-D-mannopyranoside (16)

The mannotrioside **15** (250 mg, 0.46 mmol) and Glyc_5_Boc (220 mg, 0.55 mmol) were dissolved in dry DMF (10 mL) and DCC (125 mg, 0.60 mmol), HOBt (80 mg, 0.59 mmol), and DIPEA (100 μL) added at 0 °C. The reaction mixture was stirred for 30 min at 0 °C, followed by overnight stirring at RT. The mixture was filtered through celite, the solvent reduced in vacuo and the residue purified by MPLC-RP chromatography (MeOH:H_2_O = 1:4 → 2:3) to yield, after lyophilisation, the title glycopeptide (162 mg, 0.17 mmol, 38%).

^1^H NMR (500 MHz, CD_3_OD): δ = 5.14 (d, 1H, *J*_1,2_ = 1.5 Hz, H-1), 4.93 (s, 1H, *J*_1’,2’_ = 1.6 Hz, H-1’), 4.77 (d, 1H, *J*_1’’,2’’_ = 1.9 Hz, H-1’’), 4.09, 4.05, 4.02 (each dd, each 1H, *J*_2,3_ = 3.1, *J*_2’,3’_ = 3.3, *J*_2’’,3’’_ = 3.3 Hz, H-2, H-2’, H-2’’), 3.98 (m, 8H, 3 N-C*H*_2_-CONH, manOC*H*_2_CH_2_), 3.92–3.84 (m, 6H, H-3, H-3’, H-3’’, H-6a), 3.81 (m, 4H, 2 N-C*H*_2_-CONH), 3.76 (m, 2H, manOCH_2_C*H*_2_), 3.68–3.30 (m, 9H, H-4, H-4’, H-4’’, H-5, H-5’, H-5’’, H-6b, H-6b’, H-6b’’), 1.40 (s, 9H, C(CH_3_)_3_) ppm; MALDI-TOF-MS: *m/z* 956.1 [M + Na]^+^ (955.4 calcd. for C_35_H_60_N_6_O_23_ + Na).

#### 2-[*N*-(Pentaglycyl)]-amidoethyl 3,6-di-*O*-(α-D-mannopyranosyl)-α-D-mannopyranoside hydrotrifluoroacetate (17)

The Boc-protected amine **16** (70 mg, 75.0 µmol) was stirred in aqueous TFA (90%, 1 mL) for 2 h and then co-evaporated with toluene. The residue was dissolved in water and lyophilized to yield the deprotected glycoamino acid as its TFA salt (62 mg, 64 µmol, 85%).

^1^H NMR (500 MHz, CD_3_OD): δ = 5.05 (d, 1H, *J*_1,2_ = 1.6 Hz, H-1), 4.84 (d, 1H, *J*_1’,2’_ = 1.6 Hz, H-1’), 4.79 (s, 1H, H-1’’), 4.05 (1-H, m, H-2), 4.02 (2H, s, NH-C*H*_2_-CO), 4.01 (1-H, m, H-2’), 3.98, 3.96 (each 2H, s, 2 NH-C*H*_2_-CO), 3.93 (1H, dd, H-2’’), 3.88–3.86 (m, 4H, NH-C*H*_2_-CO), 3.85–3.60 (m,19H, H-3, H-3’, H-3’’, H-4, H-4’, H-4’’, H-5, H-5’, H-5’’, H-6a, H-6a’, H-6a’’, H-6b, H-6b’, H-6b’’, manOC*H*_2_CH_2_, manOCH_2_C*H*_2_) ppm; MALDI-TOF-MS: *m/z* 855.8 [M + Na]^+^ (855.8 calcd for the free amine C_30_H_52_N_6_O_21_ + Na).

#### Bivalent target glycopeptide 1

The glycopeptide **17** (15 mg, 16 µmol) was dissolved in DMF (1 mL) and neutralized with triethylamine (8 µL). DES (3.4 µL, 23 µmol) was then added and the reaction mixture stirred at RT. According to MALDI-TOF-MS monitoring, the first ligation reaction was complete after 2 h. Then, mannoside **8** (20 mg, 32 µmol) and triethylamine (100 µL) were added and the basic reaction mixture was stirred at RT overnight. The solvent was removed under reduced pressure and the residue purified by GPC (Bio-Gel P4, water as eluent) to yield, after lyophilisation, the title compound (12 mg, 8.46 µmol, 53% based on **17**).

^1^H NMR (500 MHz, D_2_O, water suppression): δ = 4.88 (d, 1H, *J*_1,2_ = 1.6 Hz, H-1*), 4.83 (d, 1H, *J*_1’,2’_ = 1.3 Hz, H-1’*), 4.80 (d, 1H, *J*_1’’,2’’_ = 1.5 Hz, H-1’’*), 4.72 (d, 1H, *J*_1,2_ = 1.1 Hz, H-1’’’*), 4.03, 4.01, 4.00, 3.97, 3.96 (each s, each 2H, 5 NH-C*H*_2_-CO), 3.91 (m, 2H, H-2, H-2’), 3.93 (s, 2H, NH-C*H*_2_-CO), 3.88–3.81 (m, 10H, H-2’’, H-2’’’, H-3, H-3’, H-3’’, H-3’’’, H-6a, H-6a’, H-6a’’, H-6a’’’), 3.76–3.68 (m, 28H, 4 NH-C*H*_2_-CO, H-3, H-3’, H-3’’, H-3’’’, H-4, H-4’, H-4’’, H-4’’’, H-5, H-5’, H-5’’, H-5’’’, H-6b, H-6b’, H-6b’’, H-6b’’’, 2 manOCH_2_C*H*_2_, 2 manOC*H*_2_CH_2_) ppm; ^13^C NMR (125.76 MHz, D_2_O): δ = 185.45, 178.85 (C=O_SA_), 176.04, 175.43, 174.68, 174.61, 174.58, 174.44, 174.41, 174.33, 174.27, 173.82, 173.76, (C=O), 104.83, 102.08, 102.09, 101.80 (C-1, C-1’, C-1’’, C-1’’’), 75.74, 75.22, 75.12, 74.67 (C-5, C-5’, C-5’’, C-5’’’), 73.33, 73.12, 73.01, 72.92 (C-3, C-3’, C-3’’, C-3’’’), 72.55, 72.50, 72.44, 72.41 (C-2, C-2’, C-2’’, C-2’’’), 69.22, 69.16 (C-4, C-4’, C-4’’, C-4’’’), 68.10 (manOCH_2_*C*H_2_), 67.62 (manOCH_2_*C*H_2_), 64.19, 64.06, 63.69, 63.34 (C-6, C-6’, C-6’’, C-6’’’), 48.69 (manO*C*H_2_CH_2_), 46.07 (manO*C*H_2_CH_2_), 45.87 (CH_2_), 45.24 (CH_2_), 45.18 (CH_2_) ppm; assignments indexed with * are interchangable; MALDI-TOF-MS: *m/z* = 1442.2 [M + Na]^+^ (1441.5 calcd. for C_52_H_82_N_12_O_34_ + Na); ESI-MS: *m/z* 1441.1 [M + Na]^+^ (1441.5 calcd. for C_52_H_82_N_12_O_34_ +Na; ESI-HRES-MS**: ***m/z* 1441.4901 (1441.4954 calcd. for C_52_H_82_N_12_O_34_ + Na).
